# Negotiability of MB2 root canals using rotary or reciprocating systems: a randomized clinical trial

**DOI:** 10.1590/1807-3107bor-2026.vol40.049

**Published:** 2026-07-27

**Authors:** Lucas Pinto CARPENA, Lucas Peixoto de ARAÚJO, Francisco Wilker Mustafa Gomes MUNIZ, Nadia de Souza FERREIRA

**Affiliations:** (a) Univesidade Federal de Pelotas – UFPel, Dental School, Department, Pelotas, RS, Brazil.; (b) Universidade Católica de Pelotas – UCPel, Dental School, Pelotas, Rio Grande do Sul, Brazil

**Keywords:** Root Canal Preparation, Dental Instruments, Root Canal Therapy

## Abstract

This randomized clinical trial evaluated the ability to negotiate second mesiobuccal (MB2) root canals in maxillary molars using nickel-titanium (NiTi) instruments in rotary and reciprocating kinematics. A total of 110 patients with MB2 canals were randomly assigned to two groups: EL – Easy Logic2 rotary instruments (n = 57) and X1B – MKLife X1 Blue reciprocating instruments (n = 53). Canals reached to full working length (FWL) were classified as “negotiable”. In the X1B group, 96.2% of MB2 canals were negotiable, compared to 86% in the EL group, with no statistically significant difference (p = 0.152). Mean shaping time was shorter in the EL group (12.92 ± 4.36 min) than in the X1B group (14.33 ± 4.72 min), showing a significant difference (p = 0.047). Both systems showed high MB2 negotiability. However, regarding negotiability, both systems did not show statistically significant differences when adjusted Cox regression was used (Hazard ratio: 0.77; 95% confidence interval: 0.51 – 1.14). Rotary instruments showed a slight advantage in shaping time efficiency.

## Introduction

An in-depth understanding of the root canal anatomy is of utmost importance to perform proper endodontic treatment. Maxillary molars are incredibly challenging, mainly because of the presence of an extra root canal in the mesiobuccal root, called the second mesiobuccal (MB2) root canal.^1^ The lack of localization and treatment of this root canal can result in the maintenance of periapical diseases.^2^

Literature shows a high prevalence of MB2 canals in maxillary molars, which ranges from 48% to 96%.^3^ It is important to highlight that most *in vivo* studies of dental anatomy are conducted with cone-beam computed tomography images. Recent evidence reported a 90% prevalence of MB2 canals, and 77% of mesiobuccal roots presented two foramina.^4^

The localization of MB2 canals can be a difficult task. Microcomputed tomography studies have observed that the intraorifice distance between the mesiobuccal canal and the MB2 canal on maxillary molars is approximately 2.5 mm.^5,6^ Another challenge clinicians face is scouting and negotiating this root canal. One study^7^ observed that despite being apparent in 96% of cases, it was only possible to negotiate 80% of them when using the dental operating microscope and hand instruments on extracted molars.

In addition to developing different nickel-titanium (NiTi) endodontic files, a new concept in mechanized instrumentation kinematics was developed in 2008, the reciprocating movement.^8^ A previous clinical study^9^ demonstrated a greater ability of reciprocating instruments to negotiate the MB2 canal than manual instruments.

Some studies showed the superiority of mechanized instrumentation over manual instrumentation,^10,11^ resulting in more anatomically predictable root canal preparation, achievable in less time and with greater comfort for the dentist and patient alike.^12^ A variety experimental studies have been conducted to evaluate the cleaning and shaping of different reciprocating and rotary instruments.^13^

Previous *in vitro* studies have compared rotary and reciprocating kinematics in maxillary molars with MB2 canals, including evaluations of negotiation and shaping, glide path preparation, and retreatment procedures.^14-17^ These investigations have provided important insights into shaping efficiency, canal centering, and procedural errors; however, their results are limited by the absence of clinical variables. Despite the valuable information obtained from laboratory models, evidence from well-designed randomized clinical trials remains scarce.

Therefore, this randomized clinical trial aimed to compare the frequency with which rotary and reciprocating instruments reached the full working length (FWL) in MB2 canals in maxillary molars and whether there is a difference between the two systems tested. The null hypothesis tested was that there is no significant difference between both systems.

## Methods

**Figure 1:**
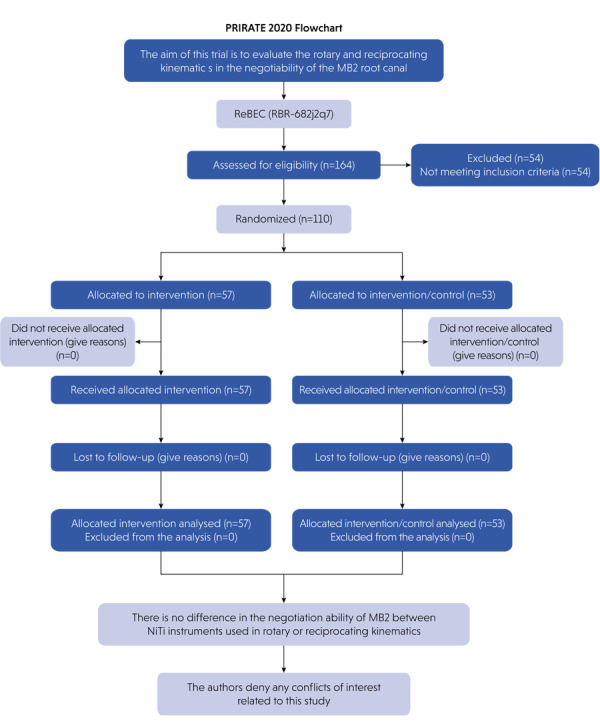
Flow diagram of patient progress at each stage of the clinical trial according to CONSORT.

This randomized controlled clinical trial is reported based on the Preferred Reporting Items for Randomized Trials in Endodontics (PRIRATE) 2020 guideline^18^ and aims to compare two different instrumentation kinematics on the negotiability of the MB2 root canal of maxillary molars regarding their effectiveness to reach the FWL. Figure 1 is a schematic flowchart that summarizes the methodological steps involved in this trial in accordance with the PRIRATE 2020. The diagram illustrates all stages of the study, including assessment for eligibility and application of the inclusion and exclusion criteria. It also depicts the randomization process, allocation of participants in groups, intervention steps, follow-up, and final analysis. This visual representation complements the text by providing a clear overview of the trial conduct and adherence to the registered protocol.

Both the rotary and reciprocating systems used in this study are manufactured from heat-treated nickel-titanium alloy, according to the respective manufacturers. The selected instruments have the same tip size but differ in taper. Rotary instrumentation was performed with Logic2 15/.05 (Easy, Belo Horizonte, Brazil) and Logic2 25/.05 (Easy), and reciprocating instrumentation was performed with X1 Blue 15/.04 (MKLife, Porto Alegre, Brazil) and X1 Blue 25/.06 (MKLife). Clinical procedures were performed by one experient endodontic specialist (LPC) who had limited his clinical activities exclusively to endodontics in the last 5 years. The operator was previously trained to standardize the procedures as well as the use of both instrumentation systems.

### Sample size calculation

From the sample size calculation to detect a difference of 10% in the mean values of negotiability, a total of 50 MB2 per group was achieved using a power of β = 80% and α = 5%. This rate was used in previous studies for this outcome.^8^ Additionally, a 10% attrition rate was added to the final sample. Therefore, 110 patients were considered necessary for the present study.

### Participant selection and ethical aspects

Patients referred for endodontic treatment in the author’s private practice between October 2019 and February 2022 were invited to participate in this study. Regarding ethical issues, this clinical trial was conducted in accordance with the Declaration of Helsinki and was approved by the Research Ethics Committee (number 3.582.440), and it was registered in the Brazilian Clinical Trials Registry under the following register code: RBR-682j2q7. The primary outcome was MB2 negotiability (ability to reach the FWL). The secondary outcome registered in the trial protocol was the occurrence of instrument fracture. Additionally, shaping time was recorded as an extra variable to provide supplementary data on procedural efficiency. This addition did not modify the original registered outcomes. After reviewing the risks, benefits, and treatment options, written free and informed consent from all patients was obtained for the proposed treatment before beginning the study.

The main inclusion criteria were patients with first or second permanent maxillary molars routinely referred for primary endodontic treatment. The exclusion criteria were patients less than 18 years old, patients with cognitive deficiencies unable to consent to the study, maxillary molar teeth requiring endodontic retreatment, teeth for which the MB2 canal was not found, access through prosthetic crowns, and cases with incomplete root formation.

### Randomization and allocation concealment

All patients were randomly assigned to each group using a computer-generated list of random numbers. Each generated number was written consecutively on white paper and placed into sealed, sequentially numbered, tamper-proof opaque envelopes by a researcher not involved in this study. The envelopes were opened by an assistant not involved in this study only at the instrumentation time, which determined the instrumentation protocol that would be performed (rotary or reciprocating kinematics). The operator knew which kinematic would be used only immediately before the mechanized instrumentation step. The patients were blinded to the kinematics used.

### Clinical interventions

**Figure 2:**
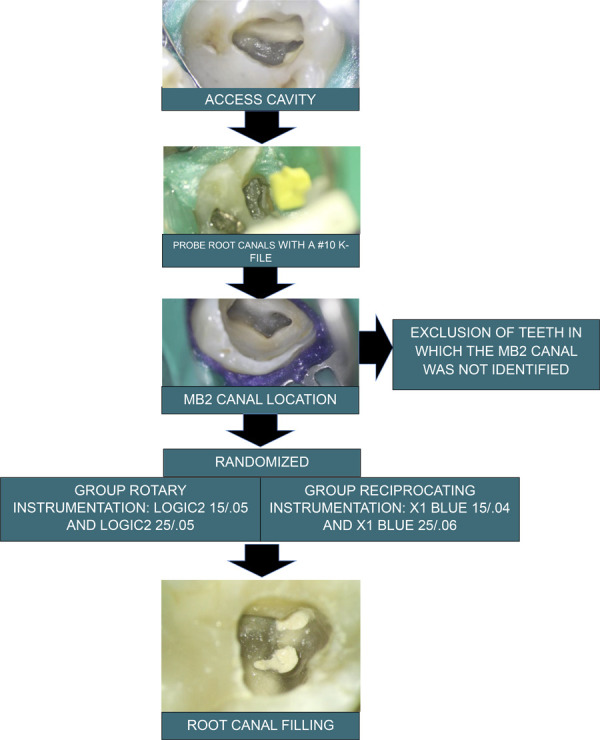
Schematic diagram of the clinical intervention and study design.

Figure 2 presents a schematic diagram of the clinical intervention performed in this study. After the proper clinical and radiographic examinations were taken and a decisive diagnosis was made for the tooth, local anesthesia was performed, and a rubber dam was placed on the referred tooth. All previous restorative materials and dental caries were completely removed with high-speed burs under magnification provided using a dental operating microscope (OPMI Pico, Zeiss, Oberkochen, Germany). Then, a traditional access cavity was made.

After obtaining access to the pulp chamber, 2% chlorhexidine gluconate gel was used to penetrate and probe the root canals with a #10 K-file (VDW, München, Germany). The protocol used for locating the MB2 was performed using E3D and E6D ultrasonic tips (Helse Ultrasonics, São Paulo, Brazil) in accordance with the American Association of Endodontics Law of Centrality for orifice location.^19^ Ultrasonic vibration was applied carefully at the junction of the tooth walls and floor in the groove between the mesiobuccal and palatal orifices. Teeth in which the MB2 canal was not identified were then excluded from this study. At this point, the patient was randomly allocated to one of the treatment groups. Initially, the mesiobuccal, distobuccal, and palatal root canals were completely cleaned and shaped before the preparation of the MB2. All mechanized files were coupled to a VDW Silver electric endodontic motor (VDW) and configured according to the manufacturer’s instructions. The instrumentation protocols for each group are described below.

#### Group 1: Rotary kinematics protocol

Negotiation of the MB2 with rotary files was performed first by carrying out coronal preflaring with the Logic2 25/.05. The instrument was driven in the electric endodontic motor according to the manufacturer’s configuration recommendation: 450 rpm clockwise and 2 n in a pecking motion with limited amplitude, cleaning its flutes with a sterile sponge every time it was removed from the root canal. The chemical auxiliary substance used was 2% chlorhexidine gluconate gel associated with bidistilled sterile water, as an irrigation substance, every time the instrument was removed. Then, FWL was determined using a #10 or #15 K-file (VDW) and an electronic apex locator (RomiApex A-15, Romidan, Kiryat Ono, Israel). After that, patency was obtained with a #10 and #15 K-file (VDW) in a watch winding motion. A glide path was performed with the Logic2 15/.05 file at 350 rpm and 1.6 n at 1 mm beyond the working length, and then, MB2 was fully prepared with Logic2 25/.05. The shaping time was recorded and registered on an Excel spreadsheet (Microsoft, Richmond, Virginia, United States). Shaping time was considered from the moment of root canal exploration with a manual #10 file until the end of the shaping at the FWL.

#### Group 2: Reciprocating kinematics protocol

The negotiation of the MB2 canal with the reciprocating kinematics protocol followed the same steps as the rotary group, except that the coronal preflaring and complete shaping of the root canal were performed with the X1 Blue reciprocating file 25/.06 and with the X1 Blue Glide Path 15/.04. Those instruments were driven in the same electric endodontic motor according to the preset configuration “Reciproc ALL”. The shaping time was also measured and registered.

## Root canal filling procedure

**Figure 3:**
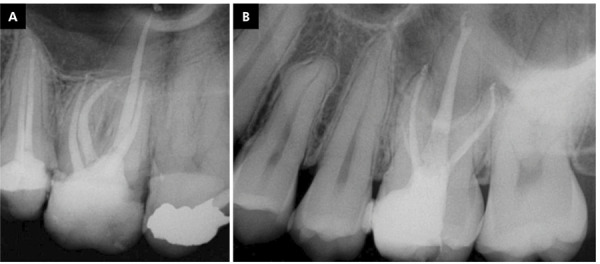
Radiograph showing the root canal filling in a case considered as negotiable (A) and a case considered as non-negotiable (B).

All root canals in both groups were filled with calibrated gutta-percha points associated with an endomethasone N root canal sealer (Septodont, Saint-Maur-des-Fossés, France). Root canal filling was performed using the single cone technique, and the calibrated gutta-percha points were cut with a heated instrument. Cases in which it was impossible to reach the FWL had the filling performed at the length the shaping instrument reached (Figure 3).

## Data recording and statistical analyses

All cases in which the rotary (G1) and reciprocating (G2) protocols reached the FWL were classified as negotiable (primary outcome). The number of MB2 classified as negotiable and nonnegotiable for both groups was tabulated. Additionally, data were collected regarding age (in years), sex (male/female), pulp vitality (yes or no), periradicular health (yes or no), and any intercurrences.

The Shapiro-Wilk test was used to test the normality of continuous variables (time to negotiate and age). Both variables demonstrated asymmetrical distributions. Therefore, comparisons between groups were performed using the Mann-Whitney test. Categorical variables were assessed using the chi-square test or Fisher’s exact test. The Mantel-Cox test was used to determine the difference in the negotiation ability of MB2 canals between the groups. The Cox proportional hazards survival regression was used to determine the influence of continuous or categorical variables on survival time with negotiability. Based on this regression, the hazard ratio (HR) and its 95% confidence interval (95%CI) were estimated, and the following covariables were selected: system (rotary group/reciprocating group), sex (male/female), age (in year), tooth type (1^st^ molar/2^nd^ molar), and tooth sensitivity (no/yes). Both crude and adjusted models were performed with all independent variables. Significance was established when p < 0.05.

## Results

This prospective clinical study included a total of 164 maxillary molars (114 first maxillary molars and 50 s maxillary molars) from 164 patients. Of the included teeth, 54 were excluded because they did not present the MB2 canal, leaving the remaining 110 teeth to be considered in this analysis. In this study, the occurrence of MB2 was observed in 78% of the first maxillary molars (n = 89) and 42% of the second maxillary molars (n = 21).

**Figure 4:**
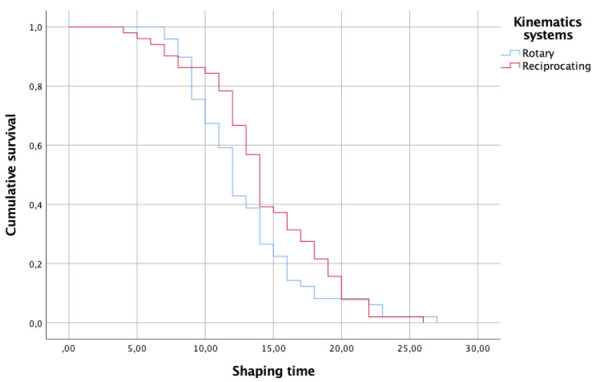
Kaplan-Meir curve of survival analysis between different systems. Survival was defined as negotiation having been achieved.

Table 1 shows the demographic and clinical data of the patients in the study groups and the main findings. The group that received the instrumentation with rotary files (n = 57) reached the FWL in 86% of the cases. Conversely, in the group of reciprocating files (n = 53), the FWL was achieved in 96.2% of the cases. Using the Mantel-Cox test, no significant differences in the ability to negotiate the MB2 were observed between the two systems (p = 0.152) (Figure 4).

**Table 1:** Demographic data from the included patients and main findings of the interventions.

Variable	Total sample	Rotary group	Reciprocating group	p-value
	(n = 110)	(n = 57; 51.8%)	(n = 53; 48.2%)	
Sex – n (%)				
Male	61 (55.5)	33 (57.9)	28 (52.8)	0.593*
Female	49 (44.5)	24 (42.1)	25 (47.2)	
Age				
Mean ± SD	39.55 ± 13.63	39.36 ± 12.85	39.75 ± 14.55	0.979**
(Median; IQR)	(38; 29.8–49.0)	(37; 29.5–48.5)	(38; 28.5–50.5)	
Tooth – n (%)				
Maxillary first molar	89 (80.9)	45 (78.9)	44 (83.0)	0.587*
Maxillary second molar	21 (19.1)	12 (21.1)	9 (17.0)	
Tooth sensitivity – n (%)				
No	51 (46.4)	28 (49.1)	23 (43.4)	0.547*
Yes	59 (53.6)	29 (50.9)	30 (56.6)	
File separation – n (%)				
No	106 (96.4)	54 (94.7)	52 (98.1)	0.619***
Yes	4 (3.6)	3 (5.3)	1 (1.9)	
Time to reach FWL (minutes)				
Mean ± SD	13.64 ± 4.58	12.92 ± 4.36	14.33 ± 4.72	0.047**
(Median; IQR)	(13; 11–16)	(12; 9.5–15)	(14; 12–18)	
Negotiability – n (%)				
No	10 (9.1)	8 (14.0)	2 (3.8)	0.061*
Yes	100 (90.9)	49 (86.0)	51 (96.2)	

*Chi-square test; ** Mann-Whitney test; ***Fisher’s Exact test; IQR Interquartile range.

Table 2 shows both the crude and adjusted analyses for the Cox regression analyses for the effect of all tested covariables with negotiability. In the crude analyses, no independent variable showed higher HR for negotiability, including the endodontic system (HR: 0.77; 95%CI 0.51–1.14). This was also observed in the adjusted analysis, as no statistically significant difference between rotary and reciprocating groups were observed (HR: 0.78; 95%CI: 0.52–1.18). Similarly, no statistically significant associations were observed for the tested covariables in both crude and adjusted models.

**Table 2:** Crude and adjusted Cox regression analyses between reaching full working length in second mesiobuccal root canals in maxillary molars and covariables. HR: harzard ratio; 95%CI: 95% confidence interval.

Variables	Crude		Adjusted	
	HR (95%CI)	p-value	HR (95%CI)	p-value
System				
Rotary group	Reference		Reference	
Reciprocating group	0.77 (0.51–1.14)	0.191	0.78 (0.52–1.18)	0.236
Sex				
Male	Reference		Reference	
Female	0.88 (0.59–1.31)	0.518	0.87 (0.58–1.31)	0.499
Age	1.00 (0.98–1.01)	0.801	1.00 (0.98–1.01)	0.793
Tooth				
First molar	Reference		Reference	
Second molar	1.24 (0.75–2.07)	0.400	1.17 (0.68–2.00)	0.576
Tooth sensitivity				
Yes	Reference		Reference	
No	1.10 (0.74–1.64)	0.630	1.07 (0.69–1.65)	0.780

File separation occurred in the MB2 root canal in both the reciprocating (n = 1) and rotary groups (n = 3), with no significant differences between groups (p = 0.619). The mean shaping time was 14.33 ± 4.72 min in the reciprocating group and 12.92 ± 4.36 min in the rotary group. A significant difference was observed in the comparison between groups regarding the shaping time, which favored the rotary kinematics group (p = 0.047).

## Discussion

This randomized clinical trial aimed to evaluate the ability to negotiate the MB2 root canal from maxillary molars using NiTi instruments in reciprocating kinematics compared with NiTi instruments in rotary kinematics. It demonstrated no significant differences between the two systems. Therefore, the null hypothesis was accepted.

In 2015, a study^9^ demonstrated the superiority of reciprocating instrumentation compared with conventional hand ﬁles in negotiating MB2 root canals. The authors discussed that reciprocating kinematics is the most decisive factor in the observed results.

In the present study, no significant difference was observed between rotary and reciprocating kinematics in MB2 negotiability to reach the FWL. The current literature is controversial in comparisons between kinematics, as there are inconclusive results regarding this issue.^21,22^ The results of *in vitro* and *in vivo* studies have shown the ability of reciprocating kinematics to advance in the root canals to the FWL, even without a glide path.^23-25^ The results of the present study also demonstrated an excellent ability of reciprocating kinematics to reach the FWL in the MB2 root canal (96.2%), but this capacity was similar to the one achieved by files in rotary kinematics (86%). Previous *in vitro* investigations have reported comparable outcomes between kinematics for glide path preparation, shaping, and retreatment of MB2 canals. These findings suggest that factors such as instrument design, taper, and alloy properties may have a greater influence on canal negotiability than the kinematic motion itself.^14-17,20^

The use of reciprocating kinematics is also suggested for narrowed and curved root canals, as it increases the life span of the instruments and improves the fatigue resistance of endodontic files compared with rotary kinematics independent of other variables, such as the speed of rotation, the angle or radius of curvature of canals, geometry, and taper, or the surface characteristics of the NiTi instruments.^26-29^ However, in the present trial, there were only four cases (3.6%) of instrument separation in the MB2 root canals. This may be explained as a glide path was performed to the FWL in all cases. The effectiveness of the glide path procedure is related to its ability to avoid the taper lock phenomenon, which stands for the binding of the instrument tip inside the root canal while its remaining bulk keeps turning.^24^

The data collected show the occurrence of the MB2 root canal in 67.1% of the first and second maxillary molars when using dental operating microscopy and ultrasonic tips to locate it. These findings are in agreement with a study that reported a mean occurrence of 73.8% of MB2 root canals in maxillary molars, with a significant variation between different populations around the globe.^3^

The literature discusses that reciprocating single-ﬁle techniques reduce the shaping time compared with rotary full-sequence systems.^13^ However, it is difﬁcult to distinguish the respective inﬂuence of different parameters, including kinematics, ﬁle design, and the number of instruments on the shaping time.

In the results of the present investigation, significantly less time was required to reach the FWL in the rotary group. These results can be partialy explained by the the expertise in clinical endodontics of the operator (LPC), and these data are in accordance with previous studies that discuss the role of the operator’s experience on the treatment quality and performance speed when using a multifile system on rotary motion comparing with reciprocating files.^30^ This result can also be explained by the fact that we did not follow the single-file technique in the reciprocating group protocol, which could be a limitation of the study.

Despite the clinical relevance of the present findings, some methodological aspects should be considered when interpreting the results. Operator blinding was not feasible because the clinician necessarily handled and visualized the instruments, which may introduce performance and detection bias. Moreover, all procedures were performed by a single experienced endodontist, which should be acknowledged as a limitation, as the results may have been influenced by individual clinical proficiency or a potential preference for one kinematic over another. However, this approach ensured strict standardization of techniques and eliminated variability from differences in skill level between clinicians. In this study, this potential bias was minimized because the operator routinely uses both systems in daily practice and underwent prior training to standardize all procedural steps. Furthermore, differences in the design and taper of the instruments used in both groups affected canal negotiability and shaping ability independently of kinematic motion, potentially confounding the interpretation of the primary outcome. While these differences reflect clinical reality, further studies should employ instruments with identical geometries and heat treatments, coupled to a motor capable of both motions, to more accurately isolate the effect of kinematics.

## Conclusion

Within the limitations of this randomized clinical trial, reciprocating and rotary kinematics demonstrated comparable effectiveness in negotiating MB2 canals in maxillary molars. The shorter preparation time observed with the rotary approach may offer an advantage in clinical practice by improving procedural efficiency without compromising canal negotiability.

## Data Availability

The anonymized dataset and SPSS syntax used for data analysis are publicly available at the Open Science Framework (OSF) under DOI: 10.17605/OSF.IO/97FT3
